# Prevalence and risk factors of deep vein thrombosis in elderly: a systematic review and meta-analysis

**DOI:** 10.1515/med-2025-1333

**Published:** 2026-01-21

**Authors:** Yajie Ren, Runbing Liu

**Affiliations:** Sinopharm North Hospital, Baotou, Inner Mongolia Autonomous Region, China

**Keywords:** deep vein thrombosis, systematic review, meta-analysis, prevalence, D-dimer, elder

## Abstract

**Objectives:**

Deep vein thrombosis (DVT) demonstrated significant health risk in elderly populations, with numerous comorbidities and biomarkers affecting its incidence. This study evaluated the prevalence, potential allied risk factors for DVT in elderly.

**Methods:**

A literature search was conducted across PubMed, Science Direct, Cochrane Library, and Google Scholar. After excluding studies, 26 studies were comprised in the study. Data were collected for DVT prevalence and associated risk factors.

**Results:**

A total of 11,651 participants comprised from China, Japan, Germany, Saudi Arabia, and Thailand. The overall DVT prevalence was 17.10 %, with higher prevalence in females (67.08 %) than males (32.92 %). Age was significantly associated with increased DVT risk (OR=1.54, 95 % CI: 0.34–2.73, p<0.01). While DVT incidence was insignificantly higher among hypertensive and diabetic patients. Significant associations identified between DVT occurrence and malignancy (OR=1.36, 95 % CI: 1.02–1.83, p<0.04), as well as recent surgical history (OR=1.34, 95 % CI: 1.01–1.77, p<0.04). Elevated D-dimer were strongly linked to DVT risk (OR=1.13, 95 % CI: 1.08–1.17, p<0.001).

**Conclusions:**

This review suggested the significant impact of aging, malignancy, recent surgery, and elevated D-dimer levels on DVT risk in elderly and recommends the assessment of clinical signs, risk factors, and biomarkers in elderly individuals.

## Introduction

DVT is a serious life-threatening pathological disorder, which is characterized through the construction of blood clots in deep veins, especially considered in the lower limbs. An approximate yearly incidence rate of DVT differs from 50 to 120 cases per 100,000 individuals in major studies performed in developed countries [1], [2], [3]. The risk of the DVT is potentially associated with age and sex. It poses significant higher risks, especially in elderly populations. Furthermore, previously reported study indicated that the annual incidence of isolated DVT in the elder population (75 or 80 years and older) often more than 400 incidences per 100,000 individuals-years, which is minimum 20 times higher than in the youngest adult population. The possible reasons for this high incidence rate in elder population might be due to age-related physiological changes, decreased physical activity and comorbidities [4], [5], [6]. DVT can produces life-threatening complications includes pulmonary embolism or stroke when it is remains as undiagnosed or untreated [7]. Therefore, an early identification of DVT is very essential to avoid serious consequences and timely interventions. However, early diagnosis of DVT is very challenging especially in the geriatric population.

The identification of key risk factors and initial clinical symptoms of DVT can help to early diagnosis of the DVT. Primary risk factors considered for DVT are elder age (>65 years), recent passed from surgery or in post-operative state, malignancy state, cardiovascular disease, metabolic abnormalities, family history related DVT or pulmonary embolism, inherited thrombophilias, inflammatory or autoimmune condition, chronic kidney disease, trauma and fracture and oral contraceptive or hormonal therapy [8]. Principal clinical features of DVT are leg pain, swelling, redness, unilateral, warmth, dilated superficial vein and leg heaviness or fatigue [9]. However, clinical symptoms of DVT can differ on basis of the location and severity of the thrombus and nonspecific symptoms are frequently overlap with other conditions such as chronic venous insufficiency, cellulitis, or lymphedema. Therefore, predictive models on basis of presence of risk factors and clinical symptoms have emerged as valuable tools to enhance early detection, risk stratification, and timely management of DVT.

Advancements in diagnostic strategies have led to the development of several clinical assessment tools includes the Wells score, the revised Geneva score, Clinical Decision Rules (CDRs) and D-dimer measurement, which help in estimating the pre-test probability of DVT [10]. These tools, when used in conjunction with ultrasonography and other imaging modalities, enhance the accuracy of diagnosis and allow for timely intervention. However, despite these advancements, the accurate identification of DVT in the elderly remains a clinical challenge, as age-related physiological changes can impact the reliability of these diagnostic methods. Therefore, the identification and assessment of crucial risk factors and biomarkers to update the parameters in the diagnostic tool is necessary. This review aims to explore and synthesize existing clinical research on prevalence and risk factors for the early diagnosis of DVT in the older age population.

## Methodology

### Data sources and literature survey

This review study has been conducted as per the “Preferred Reporting Items for Systematic reviews and Meta-Analyses”(PRISMA) guideline. A comprehensive literature survey was performed in PubMed, ScienceDirect, Cochrane and Google Scholar on 13th March 2025 through various combination of the keywords such as “deep vein thrombosis”, “DVT”, “elderly”, “older adults”, “geriatric”, “aging population”, “early detection”, “early diagnosis” and “screening”. Additional studies were identified from screened studies by cross-references. Same inclusion and exclusion criteria applied.

### Study selection

An initial screening was performed by two reviewers independently from the title and abstract as per the prior decided inclusion and exclusion criteria. Two reviewers screened the full‐text content of related studies. The view from third reviewer was taken if it is required.

### Inclusion criteria

1. Study must include elder patients (>60 years old) diagnosed with or at risk of DVT.
2. Study must evaluated the early diagnosis as risk prediction like clinical symptoms or biochemical markers for DVT.
3. Clinical trials, case-control studies, retrospective analyses and cohort studies for early detection DVT were included.
4. Study must be published in English language.

### Exclusion criteria

1. Study included non-elder population.
2. Study was not performed for early diagnosis or risk prediction.
3. Preclinical studies, cell-line studies, *in-vitro* studies and single case study were excluded.
4. Published in other than English language.

### Data collection

Two persons independently collected the data from selected study. The following items including first author, year of publication, country, study design, population type, age, total sample size, gender wise sample size, total DVT cases, gender wise DVT cases, %prevalence and gender wise %prevalence of DVT in MS Office Excel worksheet.

### Study outcome

The overall prevalence of DVT in elder population, gender wise prevalence of DVT and association of DVT incidence with different risk factors include age, BMI, hypertension, diabetes mellitus, malignancy, recent surgery, D-dimer were assessed.

### Quality assessment

The study quality of each selected article was evaluated using the Newcastle Ottawa Scale (NOS) [11], 12].

### Statistical analysis

Associations between DVT incidence and risk factors in elderly individual were analysed. A pooled odds ratio and 95 % confidence interval (CI) from the selected studies were calculated and graphically represented in a forest plot using Review Manager 5.4. p<0.05 was regarded as indicating as statistical significance.

## Results

**Figure 1:**
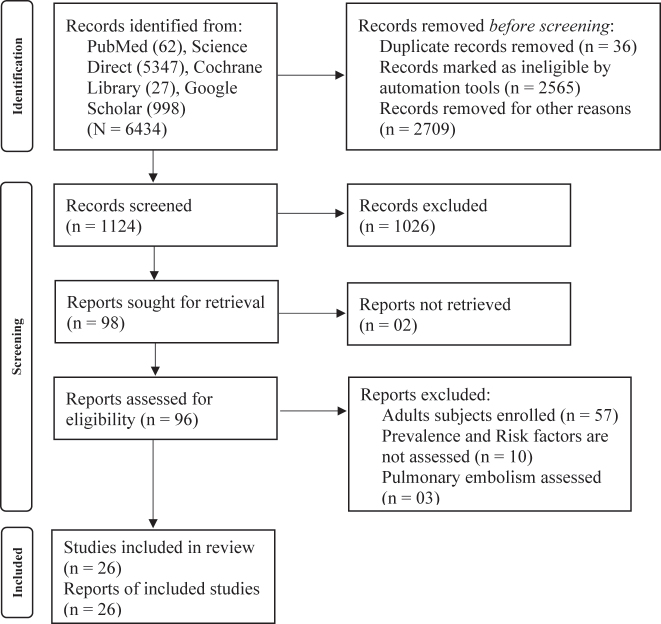
Flowchart for study selection.

The flow diagram demonstrates the search and study identification methodology. A total 6,434 studies, PubMed (n=62), Science Direct (n=5,347), Cochrane Library (n=27), Google Scholar (n=998) were identified in the initial search. A total 36 studies were identified as duplicate while 5,274 articles were removed by ineligible through automation tools and other reasons. Further, 1,026 studies were removed through title and abstract screening. Finally, 96 studies were qualified for screening of full-text content and 26 studies were lastly selected in the study (Figure-1).

The selected studies were performed at different country include China (n=21), Japan (n=2), Saudi Arabia (n=1), Germany (n=1) and Thailand (n=1). The majority of studies were performed in China and retrospective case-control studies and observational studies. In this review, total sample size was 11,651 individuals with 4,191 males 7,460 females. Amongst them, 1992 individuals were identified with DVT. The overall prevalence of DVT in elderly from 26 included studies was found to be 17.10 %. Among these, 25 studies reported the gender-specific prevalence of DVT, which demonstrated the higher prevalence of DVT in 67.08 % in female than 32.92 % in male (Table-1).

**Table 1:** Characteristics of included studies.

Sr. No.	Author	Year	Country	Type of study	Population type	Mean age (±SD)	Sample size			Total DVT cases			DVT prevalence, %		
							T	M	F	T	M	F	T	M	F
1	Xiaofei Wang	2022	China	Case-control study	Elderly intertrochanteric fractures	77.2 ± 8.5	855	312	543	105	45	60	12.3	14.4	11
2	Zhicong Wang (a)	2022	China	Case-control study	Elderly with hip fracture	–	674	238	436	128	42	86	18.99	17.65	19.72
3	Zhicong Wang (b)	2022	China	Case-control study	Elderly with hip fracture	78 + 8.6	352	124	228	53	19	34	15.06	15.32	14.91
4	Sheng Pan	2023	China	Case-control study	Elderly with hip fracture	76 (68, 83)	419	134	285	128	28	100	30.55	20.90	35.09
5	Yi-Feng Guo	2024	China	Cohort study	Elderly with end-stage osteoarthritis following total knee arthroplasty	69.2 + 5.8	1,411	241	1,170	63	9	54	4.46	3.73	4.62
6	Kitchai Luksameearunothai	2017	Thailand	Observational study	Elderly with hip fracture	78 + 10	92	24	68	15	3	12	16.30	12.50	17.65
7	Fei Xing	2018	China	Case-control study	Elderly with hip fracture	78.72 + 8.68	248	94	154	74	20	54	29.84	21.28	35.06
8	Huan Yang	2024	China	Cohort study	Elderly with hip fracture	–	74	25	49	25	7	18	33.78	28.00	36.73
9	Josef Yayan,	2016	Germany	Case-control study	Elderly	–	243	85	158	152	53	99	62.55	62.35	62.66
10	Shuai Niu	2021	China	Case-control study	Elderly with femoral neck fracture	72.5 + 8.5	980	310	670	67	22	45	6.84	7.10	6.72
11	Hiroshi Matsuo	2016	Japan	Observational study	Elderly with Acute Medical Diseases/Episodes	80.5 + 9.5	69	33	36	23	10	13	33.33	30.30	36.11
12	Reham Muqbil Alanazi	2017	Saudi Arabia	Observational study	Elderly with surgical emergency	–	540	248	292	97	57	40	17.96	22.98	13.70
13	Xiuli Cui	2024	China	Case-control study	Elderly with osteoporotic Hip Fracture	–	439	114	325	139	31	108	31.66	27.19	33.23
14	Fang Shen	2024	China	Case-control study	Elderly with PercutaneousCoronaryIntervention	–	101	48	53	44	20	24	43.56	41.67	45.28
15	Xiaorong Ma	2022	China	Case-control study	Elderly with acquired pneumonia	–	505	334	171	133	–	–	26.34	0.00	0.00
16	Chang-Song Yang	2024	China	Observational study	Elderly with hip fracture	–	223	99	124	23	10	13	10.31	10.10	10.48
17	Jiabao Jiang	2023	China	Case-control study	Elderly with hip fracture	80 + 7.9	388	117	271	134	34	100	34.54	29.06	36.90
18	Wenhao Chen	2023	China	Case-control study	Elderly with traumatic neck fracture	–	203	66	137	37	8	29	18.23	12.12	21.17
19	Shuai Han	2023	China	Observational study	Elder surgery for lower limb fracture	–	576	205	371	68	26	42	11.81	12.68	11.32
20	Li Li	2022	China	Observational study	Elderly with critically ill older adult patients	76.3 (69–84)	650	432	218	47	33	14	7.23	7.64	6.42
21	Jixing Fan	2021	China	Case-control study	Elderly with intertrochanteric fractures	78.68	788	273	515	164	47	117	20.81	9.13	42.86
22	Liang Zhang	2022	China	Observational study	Elderly with hip fracture	–	274	182	92	90	58	32	32.85	31.87	34.78
23	Yunsong Li	2024	China	Case-control study	Elderly with femoral neck fracture	72.6 + 8.2	499	153	346	47	15	32	9.42	9.80	9.25
24	Jinzeng Zuo	2020	China	Case-control study	Elderly intertrochanteric fracture	76.6 + 8.7	578	217	361	116	41	75	20.07	18.89	20.78
25	Tao Wang	2023	China	Case-control study	Eldely intertrochanteric fracture	–	318	81	237	109	16	93	34.28	19.75	39.24
26	Taizo Kaneko	2023	Japan	Case-control study	Elderly total hip arthroplasty	74 + 8.4	243	34	209	43	3	40	17.70	8.82	19.14

**Figure 2:**
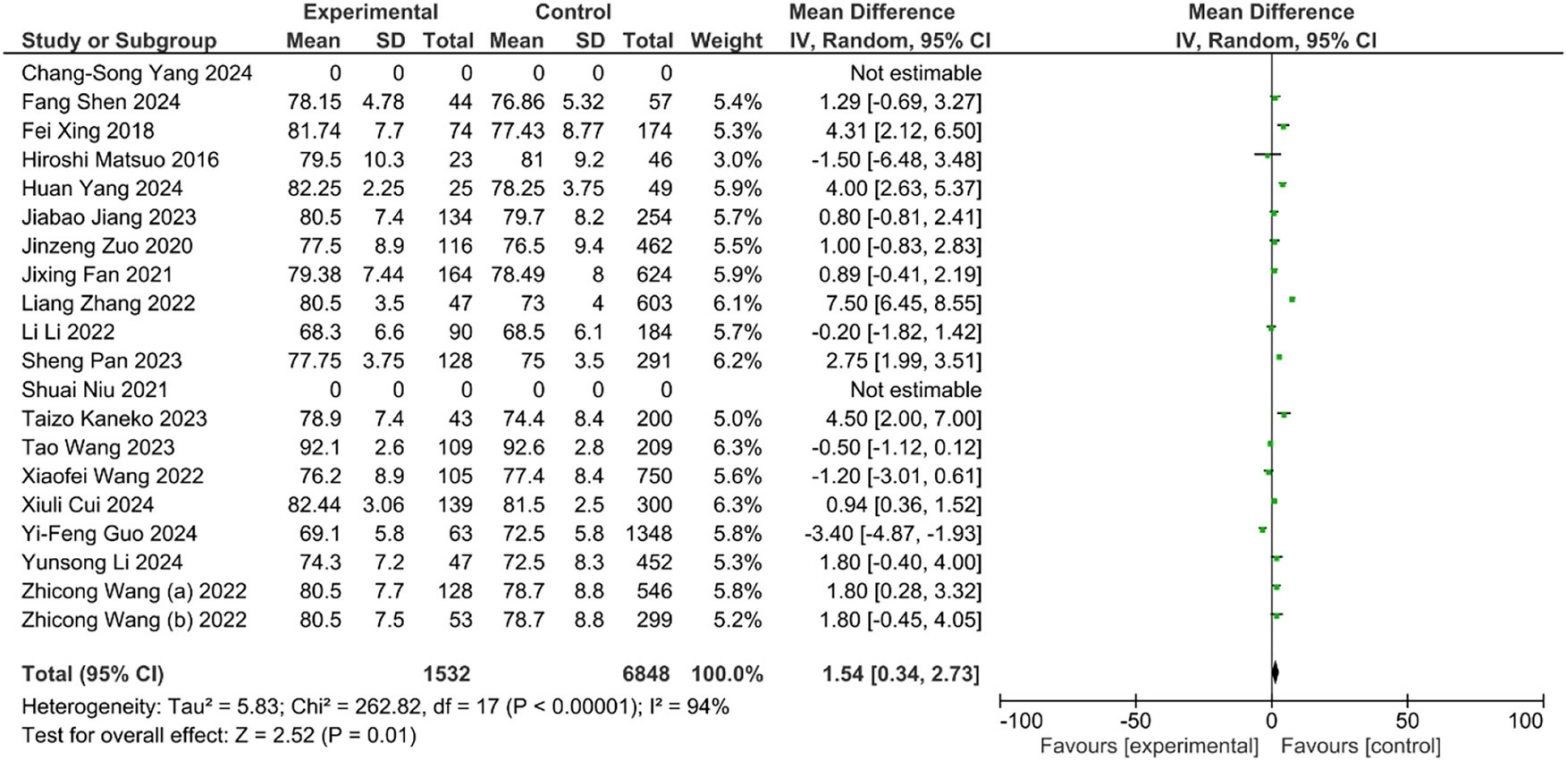
Association between age and DVT.

The results of study indicated the significantly (OR=1.54, CI: 0.34–2.73, p<0.01) association between increasing of age and incidence of DVT. It is suggesting the higher risk of DVT in elder population than younger adults (Figure-2).

**Figure 3:**
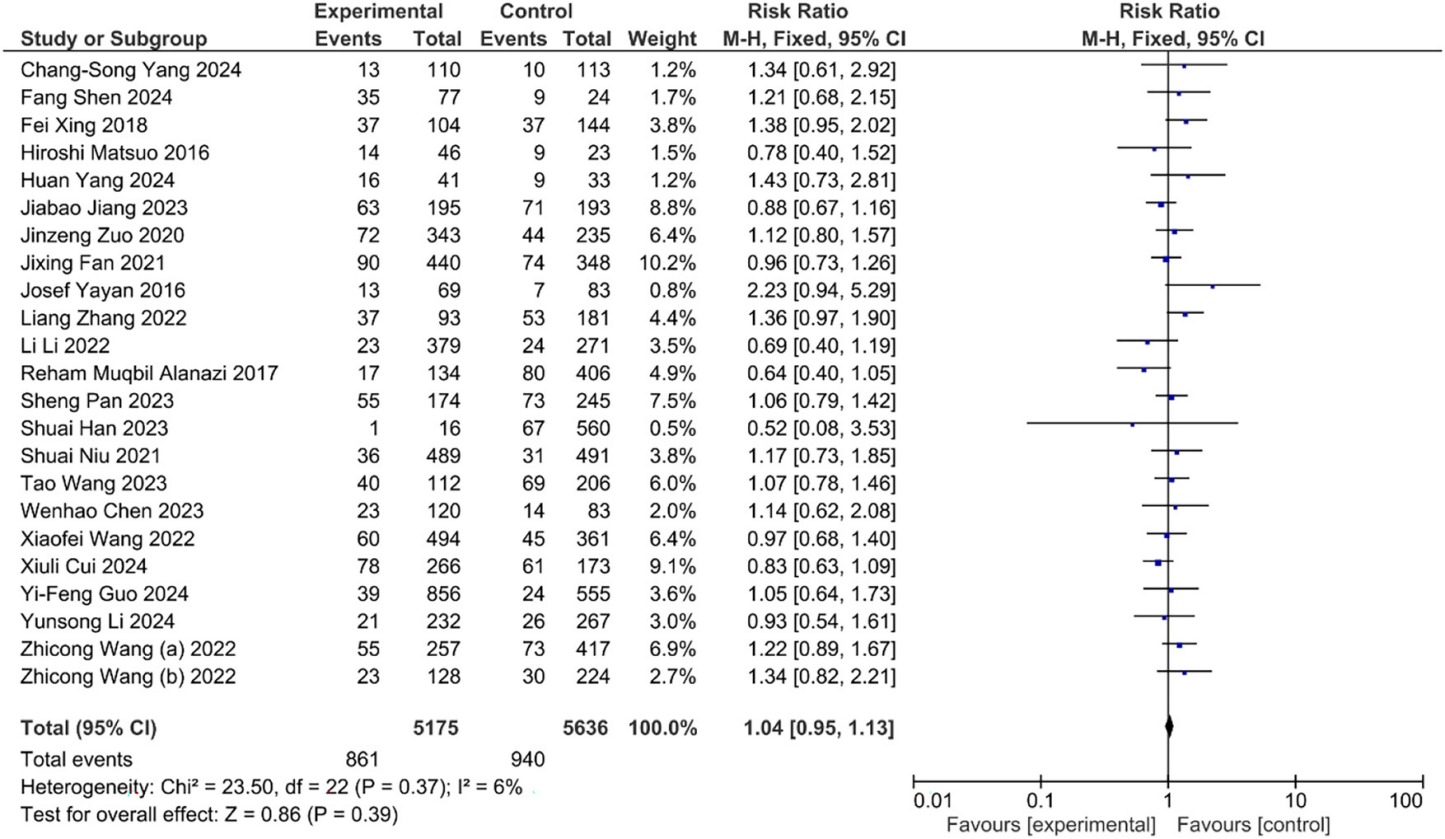
Association between hypertension and DVT.

**Figure 4:**
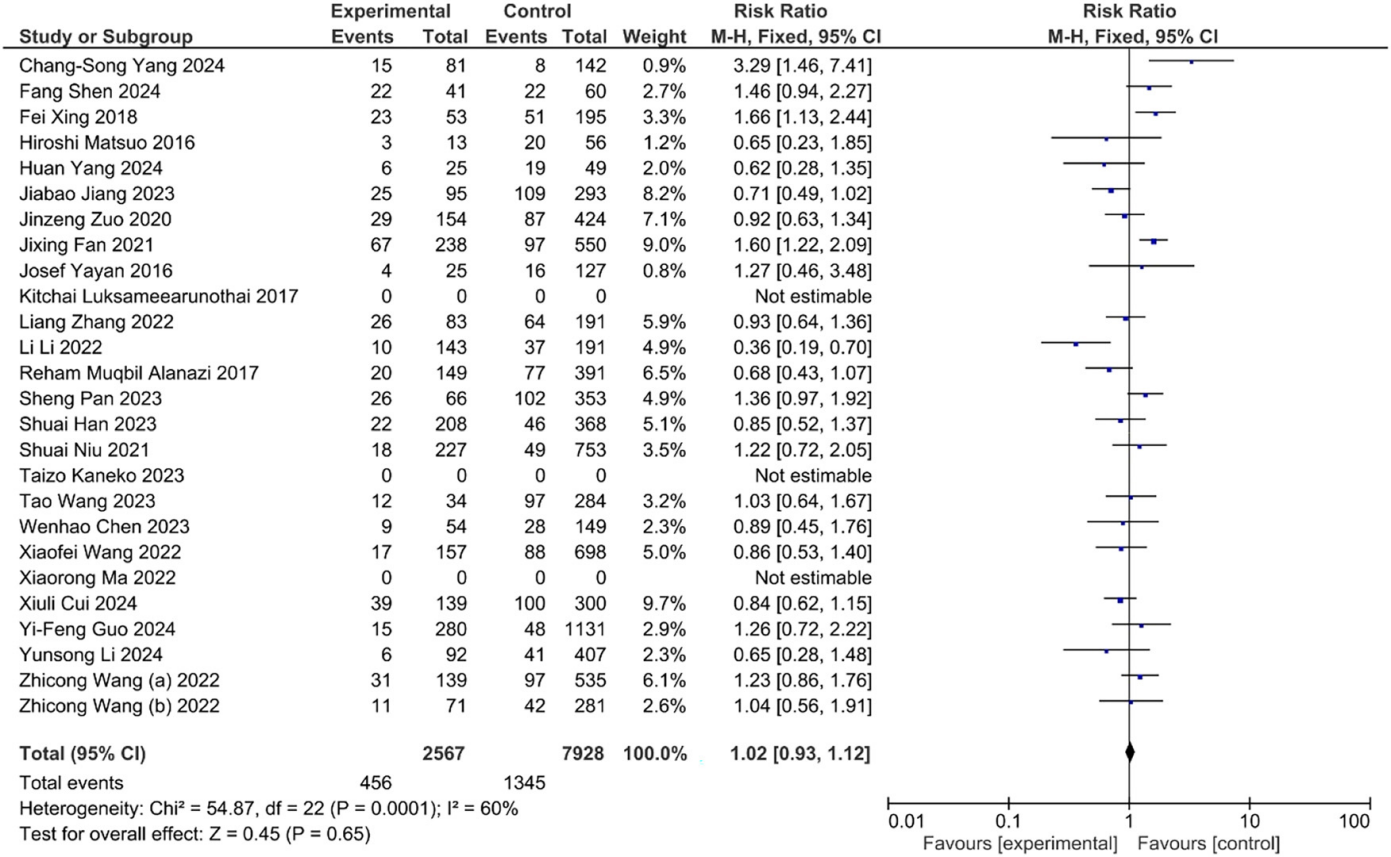
Association between diabetes and DVT.

The incidence of DVT indicated higher in the hypertensive individual as compared to normotensive individual. Although, this association was statistically insignificant (OR=1.04, CI: 0.95, 1.13, p<0.39) (Figure-3). Similarly, the incidence of DVT had higher in the diabetic mellitus patients as compared to non-diabetic. However, this association was insignificant statistically (OR=1.02, CI: 0.93, 1.12, p<0.65) (Figure-4).

**Figure 5:**
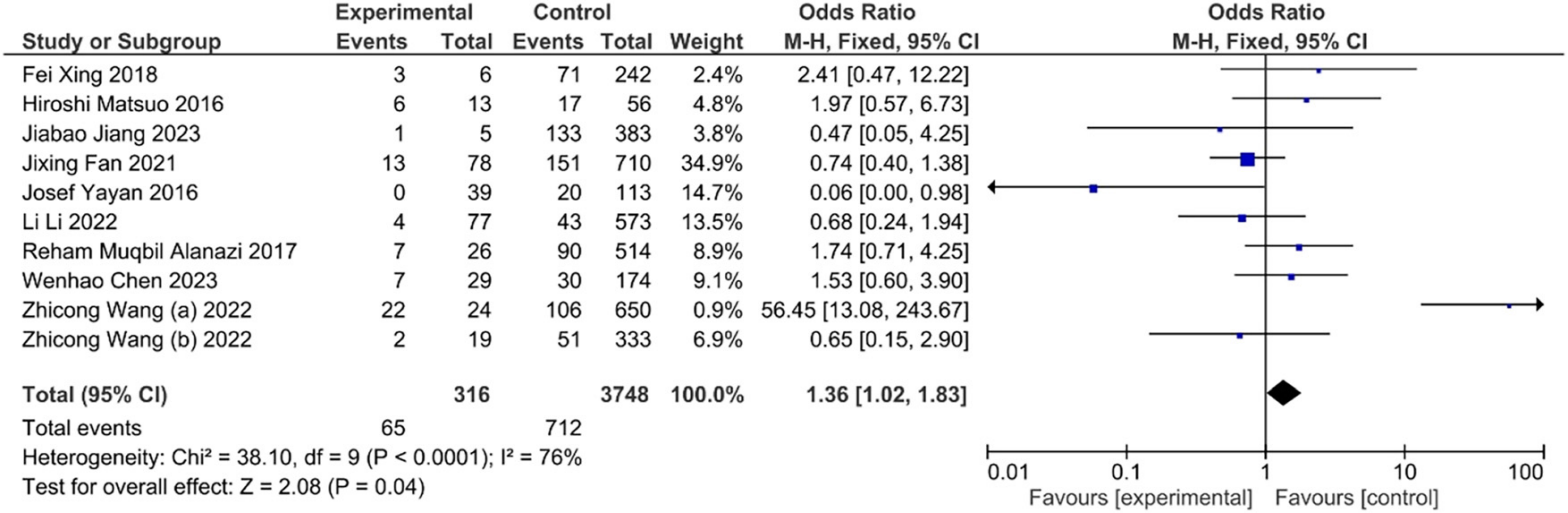
Association between malignancy and DVT.

**Figure 6:**
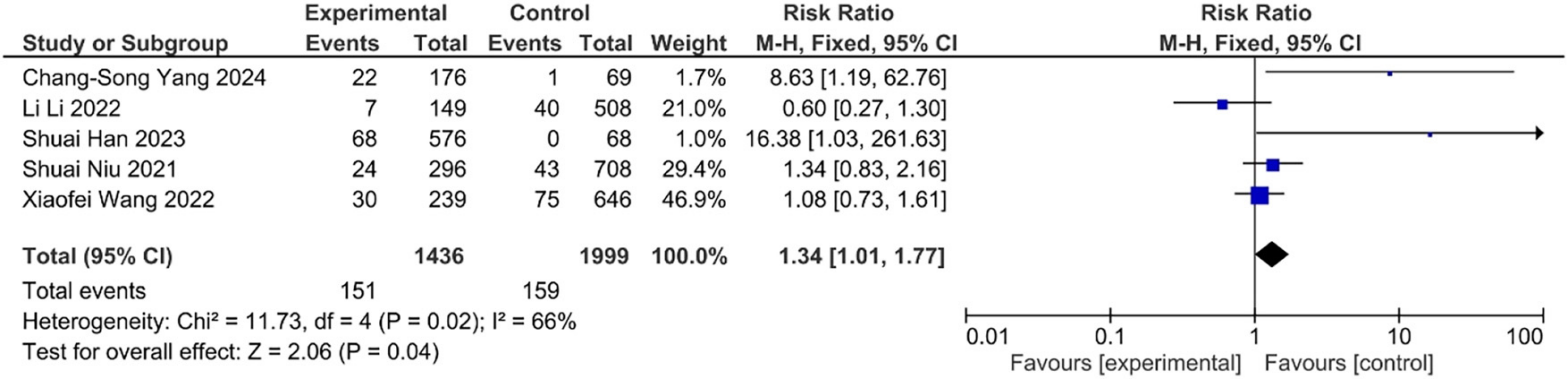
Association between recent surgery and DVT.

The occurrence of DVT was significantly higher (OR=1.36, CI: 1.02, 1.83, p<0.04) with malignancy and suggested the crucial association between risk of DVT and cancer (Figure-5). Likewise, the occurrence of DVT was significantly higher (OR=1.34, CI: 1.01, 1.77, p<0.04) in individual having history recent surgery (Figure-6). This association suggested the surgical intervention as potential risk factor for DVT.

The elevated D-dimer levels were associated with increased odds of DVT (OR=1.13, 95 % CI: 1.08–1.17, p<0.001) (Figure-7A). Furthermore, subgroup analysis demonstrated that DVT patients were significantly more likely to exhibit D-dimer levels>0.5 mg/mL compared to non-DVT individuals (OR=1.40, 95 % CI: 1.02–1.93, p<0.04) (Figure-7B). This association suggested the D-dimer level as potential biochemical marker for DVT risk.

**Figure 7A:**
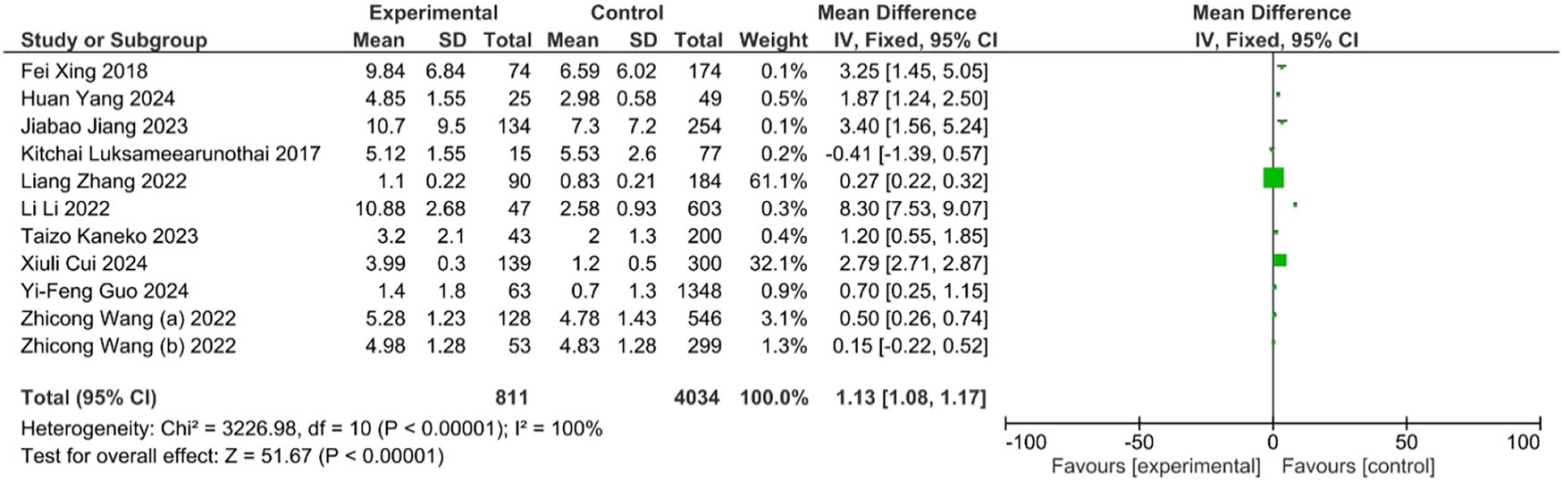
Association between D-dimer level and DVT.

**Figure 7B:**
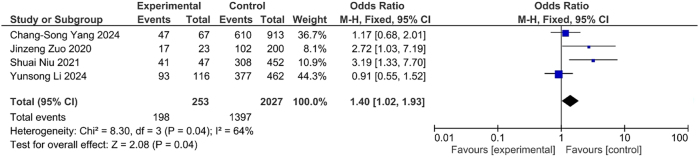
Association between elevation of D-dimer and DVT.

## Discussion

This review study evaluated the prevalence and risk factors associated with DVT among the different elderly population, focusing on clinical variables such as age, hypertension, diabetes mellitus, malignancy, recent surgical history, and D-dimer levels as biomarker. This study demonstrate the comprehensive synthesis of 26 studies and highlight many vital associations, which have significant implications for risk minimization and clinical management of DVT in high-risk populations.

The overall prevalence of DVT in the elderly population across the included studies was found to be 17.10 %, confirming that DVT remains a significantly higher health burden in elderly population. Furthermore, a pooled odds ratio confirmed that this association among older age and increased DVT risk is statistically significant.

These results are consistent with prior reported studies, representing that aging is one of the robust risk factors for venous thromboembolism (VTE), including DVT. The cases of VTE twice with each decade after age 50, highlighting the influence of vascular aging, decreased mobility, and pro-thrombotic changes in the coagulation cascade in the elderly [13],^14^]. A large European registry suggested the higher incidence of DVT as compared to PE in the 80–84 elderly population [15]. Immobility is also considered as one of the most crucial factor for enhancing the risk of thrombotic events irrespective to age of individual [16], 17]. Furthermore, comorbidities like chronic inflammation, heart failure, and cancer are frequently present in older populations, which can exaggerate the risk DVT [18], 19].

Among 25 studies that reported gender-specific data, the prevalence of DVT was 32.92 % in males and 67.08 % in females. This results suggested the possible impact of hormonal or physiological changes. Many studies reported the exposure to estrogen enhanced the risk of thrombotic events whether endogenous or through hormone replacement therapy mainly in postmenopausal women [20],^21^]. Although, further studies are required to elucidate whether these results indicate true biological differences or are confounded by other clinical or demographic factors.

Hypertension is a very commonly observed as comorbidity in elderly and a known as principal contributor to vascular dysfunction. The results of this study suggested hypertensive individuals had a slightly higher incidence of DVT but the association was statistically insignificant. Similarly, the presence of diabetes mellitus also showed insignificantly increase the odds of DVT. These findings contrast with some prior studies suggesting a pro-thrombotic state in hypertensive and diabetic individuals due to endothelial dysfunction, increased platelet activation, and inflammation [22], 23]. Many single study reported the inconsistent findings on the risk of DVT associated to diabetes mellitus [24], [25], [26]. The DVT risk allied to diabetes mellitus was indicated the more in younger population as compared to elderly [24]. The lack of significant associations in our meta-analysis could be attributed to heterogeneity in diagnostic criteria, study populations, or the presence of confounding variables not fully accounted for in the primary studies.

A strong associations identified between DVT and malignancy. These results are consistent with well-established clinical evidence indicating that cancer and surgery are two of the most strong developed risk factors for DVT [20]. It has been suggested the overall risk of DVT in malignancy is seven times more compared to non-malignancy [27]. Malignancies, specifically those having pancreas, lung, and gastrointestinal tract, are known to promote a hypercoagulable state through tumor-cell–induced tissue factor expression and pro-inflammatory cytokine production [28]. These findings strengthen the available clinical guidelines that recommend prophylaxis for thrombotic events in malignancy patients, cardiovascular disease and perioperative patients undergoing major surgery [29], [30], [31].

In this study, potent association identified between DVT and recent surgery. Surgical procedures, particularly those involving prolonged immobilization or orthopaedic intervention significantly increased the DVT risk by causing vascular injury and venous stasis [32], 33]. In the context of surgery-related thrombogenesis, immobilization serves as a principal contributor to the development of DVT by promoting venous stasis and impairing normal blood flow dynamics. Consequently, the history of postoperative bed rest is incorporated as a critical parameter in the primary assessment criteria, given its direct mechanistic association with thrombus formation. The occurrence of DVT following surgery is follows a temporal pattern influenced by surgical trauma, perioperative immobility, systemic inflammation, and patient-specific risk factors [34]. The study of the different time interval for development of DVT after surgery may provide the greater precision for assessing the DVT risk, development of effective prophylactic strategies and better postoperative outcomes [35], 36].

This analysis showed that increased D-dimer level significantly associated with DVT incidence. Furthermore, the presence of D-dimer levels above 0.5 mg/mL was significantly higher observed in DVT individuals as compared to non-DVT individuals. D-dimer is a produced from degradation of fibrin, has extensively been documented as a valuable biomarker for ruling out DVT as it has high sensitivity [37]. However, its specificity is limited, especially in elderly, where baseline levels may be elevated due to age-related inflammation, comorbidities, or recent surgeries [38]. In such condition, incorporation of additional molecular markers such as P-selectin, glycoprotein IIb/IIIa complex, VEGF, sE-selectin, PAFR, genetic mutation (factor II and factor V Leiden) may provide the higher accuracy to identify the DVT risk in patient. Nonetheless, the statistical significance observed in this study supports the continued use of D-dimer as a diagnostic tool, particularly in combination with clinical signs, other markers and presence of risk factors.

While the study was provided a comprehensive overview of DVT risk factors in the elderly, certain limitations should be acknowledged. First, heterogeneity in study design, population characteristics, and diagnostic criteria may have influenced the pooled estimates. Second, not all studies reported adjustments for confounders, which may limit the causal interpretation of the associations. The majority of the included studies were conducted in China hence the findings may primarily reflect the clinical practices and patient characteristics of that region, thereby potentially limiting their generalizability to other populations. Lastly, the reliance on observational data limits our ability to infer temporal relationships.

The findings of this systematic review may helpful to risk stratification include older age, malignancy, recent surgery, and increased D-dimer levels must be recognized high-risk marker in elderly patients presenting with leg swelling, pain, or immobilization. Incorporating D-dimer as important biomarker into standard diagnostic algorithms may enhance early identification and reduce unnecessary imaging, particularly in low-risk populations.

## Conclusion

In conclusion, this meta-analysis confirmed that age, malignancy, recent surgery, and increased D-dimer levels were significantly associated to enhancement of DVT risk in elderly individuals. While hypertension and diabetes showed a trend toward increased incidence. These findings strengthen the importance of tailored risk assessment and preventive strategies in high-risk populations, mainly in settings where initial identification and prophylaxis can significantly decrease morbidity and fatality associated with DVT.
